# What Is the Interversion Reliability and Agreement Between the Decision Tree Patient-rated Wrist Evaluation and the Full-length Version?

**DOI:** 10.1097/CORR.0000000000003933

**Published:** 2026-05-08

**Authors:** Sebastiaan Tjènne Peters, Robbert Maarten Wouters, Ruud Willem Selles, Harm Pieter Slijper

**Affiliations:** 1 Department of Plastic, Reconstructive and Hand Surgery, Erasmus MC, University Medical Center Rotterdam, Rotterdam, the Netherlands; 2 Department of Rehabilitation Medicine, Erasmus MC, University Medical Center Rotterdam, Rotterdam, the Netherlands; 3 Hand and Wrist Center, Xpert Clinics, Eindhoven, the Netherlands; 4 Center for Hand Therapy, Xpert Handtherapie, Utrecht, the Netherlands

## Abstract

**Background:**

A frequently used strategy to improve patient-reported outcome measure (PROM) response rates and reduce patients’ burden with PROM completion is item reduction. In this context, a shortened decision tree version of the Patient-Rated Wrist Evaluation (DT-PRWE) was developed, reducing the number of items from 15 to 5. The DT-PRWE demonstrated excellent psychometric properties in simulated data; however, its psychometric properties have not yet been evaluated in a real-world clinical setting.

**Questions/purposes:**

(1) What is the interversion reliability and agreement between the DT-PRWE and the Patient-Rated Wrist Evaluation (PRWE) in a clinical setting? (2) What is the difference in completion time between the DT-PRWE and the PRWE?

**Methods:**

We conducted a prospective study at Xpert Clinics in the Netherlands, a multicenter, referral-based outpatient practice, with both urban and regional locations, that specializes in hand and wrist surgery and hand therapy to assess the interversion reliability and agreement between the PRWE and the DT-PRWE. Between January and April 2025, a total of 427 adult patients were treated for wrist-related conditions and completed the PRWE at baseline as part of routine outcome measurement at our clinic. Subsequently, we asked patients to complete the DT-PRWE again 5 to 10 days after the initial assessment. Of those, we considered adult patients with wrist conditions who completed both versions of the PRWE to be potentially eligible. Based on this, 55% (235 of 427) were potentially eligible; a further 27% (116) were excluded because of intervening treatment before completion of the PRWE and the DT-PRWE, including corticosteroid injection (11% [45]), surgery before completion of both versions (6% [25]), other treatment before completion of both versions (5% [23]), and concomitant treatment (5% [23]), leaving 28% (119) for analysis here. Primarily, we evaluated interversion reliability using intraclass correlation coefficients (ICC) as the main outcome; an ICC > 0.75 was considered acceptable for clinical use. We also calculated Pearson correlation coefficients. We assessed the agreement by evaluating paired between-version mean differences, standard error of measurement (SEM), and Bland-Altman plots. Additionally, we compared the SEM values with the minimum important change (MIC) thresholds of the PRWE to assess the level of agreement. Finally, we calculated the median (IQR) completion time and compared completion efficiency between versions using the paired Wilcoxon signed-rank test.

**Results:**

The DT-PRWE demonstrated good interversion reliability compared with the PRWE for total score (ICC 0.88 [95% confidence interval (CI) 0.83 to 0.91]), pain subscore (ICC 0.78 [95% CI 0.69 to 0.84]), and hand function subscore (ICC 0.83 [95% CI 0.77 to 0.88]). Additionally, the scores of the PRWE and DT-PRWE were highly correlated (total score r = 0.88 [95% CI 0.83 to 0.92], pain subscore r = 0.81 [95% CI 0.74 to 0.87], hand function subscore r = 0.83 [95% CI 0.77 to 0.88]). The agreement between versions was high, with between-version mean differences of -5.3 (95% CI -7.2 to -3.5) on the total score (score range 0 to 100), -3.4 (95% CI -4.6 to -2.2) on the pain subscore (score range 0 to 50), and -2.0 (95% CI -3.2 to -0.7) on the hand function subscore (score range 0 to 50). The SEM values were 7.1 for the total score, 4.6 for the pain subscore, and 4.9 for the hand function subscore, all falling below the MIC thresholds. The Bland-Altman plots indicated high agreement. The median (IQR) time to complete the PRWE was 3 minutes 33 seconds (2 minutes 20 seconds to 7 minutes 18 seconds), whereas for the DT-PRWE it was 1 minute 5 seconds (50 seconds to 1 minute 29 seconds), representing a 70% reduction with a median difference of 2 minutes 28 seconds (p < 0.001).

**Conclusion:**

The DT-PRWE is a reliable alternative to the full-length version, requiring substantially less time for patients to complete. While preserving both pain and function subscores, its simple digital implementation and comparability with existing PRWE data make the DT-PRWE well suited to replace the PRWE in routine clinical practice and for research applications. Future research should focus on cross-cultural validation of the DT-PRWE and test-retest reliability. Also, future research may investigate whether implementing shortened PROMs, such as the DT-PRWE, improves compliance with PROMs.

**Level of Evidence:**

Level I, diagnostic study.

## Introduction

In hand and wrist care, a commonly used patient-reported outcome measure (PROM) is the Patient-Rated Wrist Evaluation (PRWE), a region-specific instrument designed to assess wrist pain and hand function in patients with wrist disorders that shows strong psychometric properties [16, 29, 30]. The PRWE consists of two subdomains (pain and hand function), with their scores combined to yield a total score [16]; however, despite the clinical value of the PRWE and other PROMs, achieving high response rates remains challenging [20, 23]. A common strategy to improve response rates and reduce patients’ burden when completing PROMs is item reduction [1, 5].

In this context, a fixed decision tree version of the PRWE (DT-PRWE) was previously developed, reducing the number of items from 15 to 6 (three per subdomain) [26]. The DT-PRWE uses a fixed decision tree with conditional logic to omit questions that are irrelevant to a patient’s symptoms. Based on responses to earlier items, subsequent questions are either presented or skipped, allowing fewer items to be completed while retaining coverage of both PRWE subdomains [8, 12, 26]. This fixed structure can be implemented within standard electronic PROM collection systems. The DT-PRWE demonstrated excellent interversion reliability and agreement on a validation data set that simulated responses based on the original PRWE, with intraclass correlation coefficient (ICC) values exceeding commonly accepted thresholds for good reliability [26].

Although the DT-PRWE has the potential to increase response rates and reduce patients’ burden, it has not yet been adopted in routine clinical practice or research because its psychometric properties lack external validation. This step is essential to confirm that the decision tree version is not only reliable in controlled environments—where responses are simulated from the PRWE—but also that it is feasible, accurate, and acceptable in everyday care. Demonstrating consistency between versions in daily practice would strengthen the evidence for using the DT-PRWE, aiding wider adoption and ultimately supporting clinicians in delivering value-based healthcare while reducing patient burden.

We therefore asked: (1) What is the interversion reliability and agreement between the DT-PRWE and the PRWE in a clinical setting? (2) What is the difference in completion time between the DT-PRWE and the PRWE?

## Patients and Methods

### Study Design and Setting

We conducted a psychometric study to evaluate the interversion reliability and agreement between the DT-PRWE and the PRWE. We reported the results in accordance with the guidelines of the Strengthening the Reporting of Observational Studies in Epidemiology (STROBE) statement [28].

We collected data prospectively at Xpert Clinics in the Netherlands, a nationwide, multicenter referral-based outpatient practice with both urban and regional locations that specializes in hand surgery and therapy. Data were collected from January to April 2025 using the GemsTracker electronic data capture system (Erasmus MC, Equipe Zorgbedrijven; https://gemstracker.org). Xpert Clinics has 28 clinics staffed by fellowship-trained or Federation of European Societies for Surgery of the Hand (FESSH)–certified hand surgeons (experience levels III to V [24]) and more than 150 specialized hand therapists. After their initial consultation, patients with wrist conditions were invited to complete the Dutch version of the PRWE as part of routine outcome measurement, in accordance with the International Consortium for Health Outcomes Measurement (ICHOM) standard set for hand and wrist [6, 23, 27, 29]. In our routine clinical practice, many patients do not receive immediate treatment during the initial consultation, as interventions such as orthosis or hand therapy are often initiated at a subsequent visit with our hand therapists, and surgical treatment is usually scheduled at a later date. This workflow allowed assessment of both the PRWE and DT-PRWE at separate time points, thereby limiting recall effects and avoiding potential changes in symptoms attributed to intervening treatment, which could otherwise compromise comparability between versions. PROMs at our clinic are administered digitally via the GemsTracker system, an electronic data capture platform that integrates LimeSurvey, an online survey tool. GemsTracker automatically sent email invitations to patients and allowed them to complete PROMs independently, without a clinician’s presence or involvement. Subsequently, we asked patients to complete the DT-PRWE within a 5- to 10-day window after completing the PRWE.

### Patients

Patients were eligible for inclusion if they (1) completed both the full-length and decision tree versions of the PRWE within 5 to 10 days, (2) were older than 18 years at the time of completion, and (3) had a diagnosis of a wrist-related condition. Patients were excluded if they (1) received treatment (for instance, surgery, orthosis, and/or hand therapy) before completing both versions, as this could alter symptoms and affect comparability, (2) received a corticosteroid injection as a treatment, as this has an immediate effect on symptoms, or (3) underwent concomitant treatment, which could confound the assessment of interversion reliability and agreement. We reviewed patient records manually to assess the exclusion criteria.

**Fig. 1:**
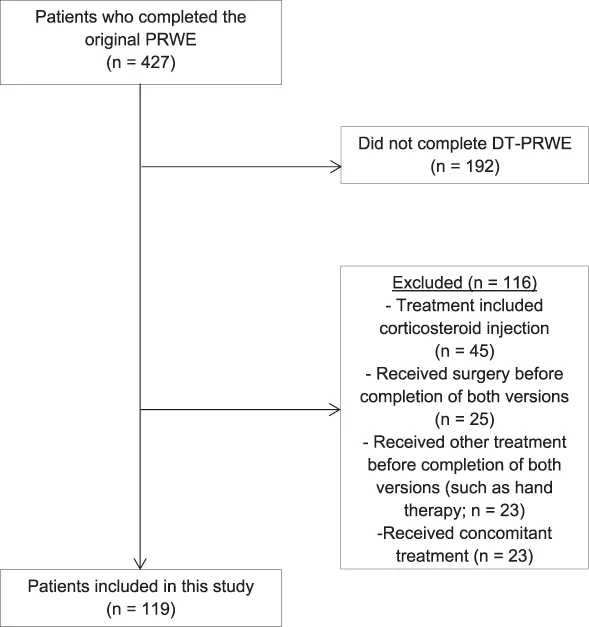
Flow chart of the selection of patients included in this study.

During the study period, 427 patients completed the PRWE, and we subsequently invited these patients to complete the DT-PRWE 5 days after completing the full-length version. Of these patients, 235 completed the DT-PRWE. After applying the eligibility criteria, we were able to include 119 patients in our study (Fig. 1).

### Descriptive Data

Of these 119 patients, 39% (47) were men, 61% (72) underwent surgical treatment, and the mean ± SD age was 53 ± 17 years. The median (IQR) number of days between completion of both versions was 5 (5 to 6). The most common diagnoses were scapholunate ligament lesion (17% [20]), scapholunate advanced collapse or scaphoid nonunion advanced collapse of the wrist (12% [14]), triangular fibrocartilage complex lesion (18% [21]), wrist instability (13% [15]), and wrist osteoarthritis (20% [24]) (Table 1).

**Table 1.** Characteristics of the included patients

Variable	Value (n = 119)
Age in years	53 ± 17
Gender, men	39 (47)
Hand dominance	
Ambidextrous	9 (11)
Left	12 (14)
Right	79 (94)
Duration of symptoms in months	6 (4-12)
Delta days between completion	5 (5-6)
Type of work	
Retired	19 (22)
Unemployed	11 (13)
Light	29 (34)
Moderate	23 (27)
Heavy	19 (23)
Education level	
Primary education	1 (1)
Lower secondary education	13 (16)
Upper secondary education	26 (31)
Postsecondary vocational education	28 (33)
University	29 (34)
Prefer not to say	3 (4)
Surgical treatment	61 (72)
Diagnosis	
Carpal fracture (scaphoid or hamate)	5 (6)
Flexor tendon synovitis of the wrist	3 (3)
Kienböck disease	1 (1)
Malunion of the radius or ulna	3 (3)
Scapholunate ligament lesion	17 (20)
SLAC/SNAC of the wrist	12 (14)
Subluxation sixth extensor compartment	3 (3)
Triangular fibrocartilage complex lesion	18 (21)
Ulnocarpal abutment	8 (9)
Wrist instability	13 (15)
Wrist osteoarthritis	20 (24)

Data presented as mean ± SD, % (n), or median (IQR). SLAC/SNAC = scapholunate advanced collapse or scaphoid nonunion advanced collapse.

### Variables and Measurements

The PRWE has two subdomains that measure pain and discomfort during daily activities (5 items) and wrist and hand function (10 items). Both subdomain scores range from 0 to 50, with a lower score indicating better performance. Each item is rated on a 10-point Likert scale, with 0 representing no pain or difficulty with hand function and 10 indicating the worst pain, or that the activity cannot be performed, for the pain and hand function subdomains, respectively. The pain subscore is calculated by totaling the five pain items (range 0 to 50), whereas the hand function subscore (range 0 to 50) is obtained by summing the 10 items and dividing by 2. Both subscores are combined to form a total score from 0 to 100. The total score is the sum of the pain and hand function subscores (range 0 to 100) [16].

**Fig. 2:**
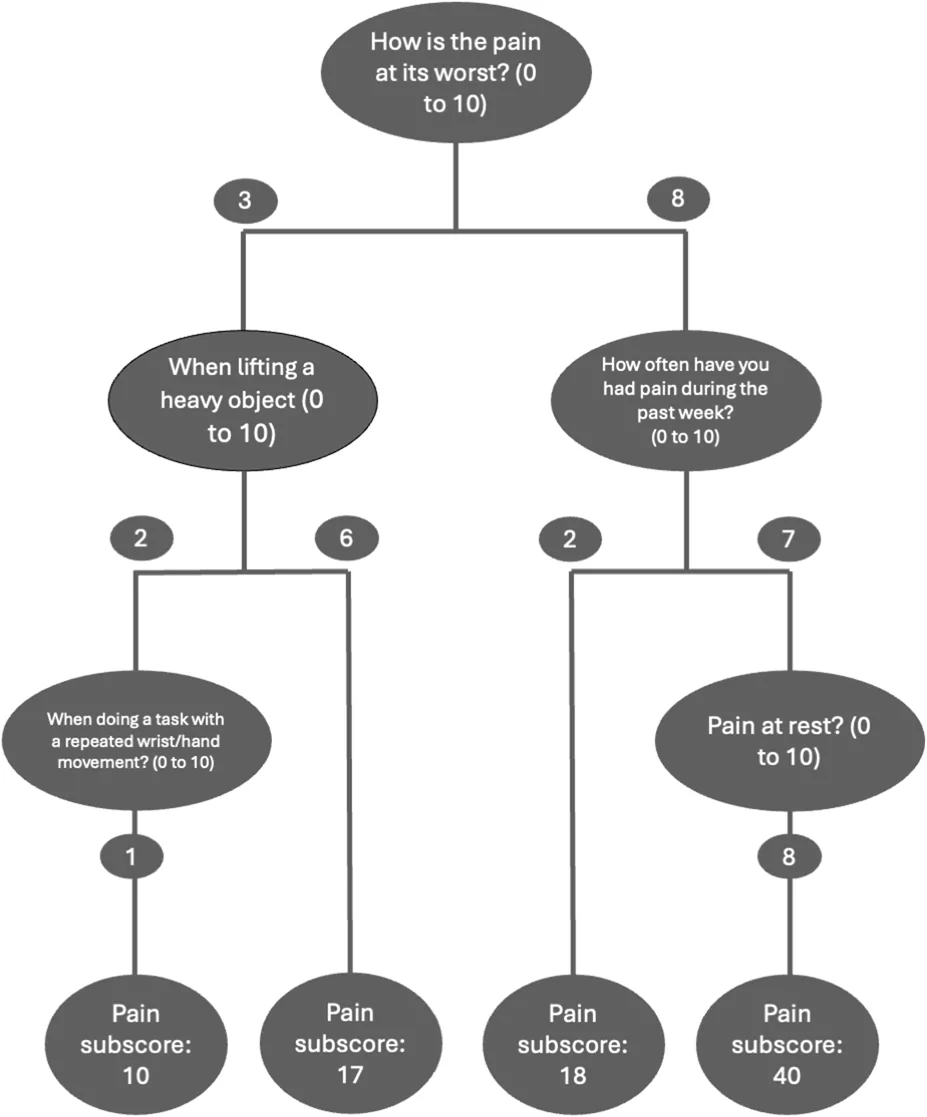
This figure illustrates a simplified version of the pain subscore fixed decision tree, demonstrating four example response paths through the decision tree. The numbers in the ovals represent example patient responses (0 to 10) to each item, which determine the subsequent follow-up question according to the decision tree. An estimated pain subscore is assigned when an end node is reached.

The DT-PRWE includes the same subdomains and items as the PRWE, starting with the pain subdomain at item 4 (when the pain is at its worst) and the hand function subdomain at item 8 (difficulty with household tasks, such as cleaning and maintenance). Based on the responses, subsequent items are selected until, using a fixed decision tree (Fig. 2), a final subscore is determined for each subdomain. A maximum of three items (range 2 to 3) per subdomain are needed to generate the estimated subscores (range 0 to 50). The total score is calculated by summing the two subscore estimates (range 0 to 100) [26].

### Study Size

We performed a sample size calculation for the ICC assuming a null hypothesis ICC of 0.50 (moderate reliability) and an expected ICC of > 0.75 (good reliability) [13], with α = 0.05, power = 0.80, and two measurements per subject (DT-PRWE and PRWE). Based on this calculation, a minimum of 45 participants was required. In addition, the Consensus-based Standards for the Selection of Health Measurement Instruments (COSMIN) guidelines [19] recommend a minimum sample size of 100 patients to calculate the ICC, which is lower than the 119 patients included in this study.

### Ethical Approval

The medical research ethics committee of Erasmus MC, Rotterdam, approved our study (MEC-2024-0540), and written informed consent was obtained from all patients.

### Statistical Analysis

We assessed the interversion reliability by calculating the ICC as the primary outcome. An ICC of 0.75 was considered acceptable for clinical use, as this is a common threshold for good reliability [13]. Additionally, we calculated Pearson correlation coefficients between both versions. A Pearson correlation coefficient of ≥ 0.70 was considered acceptable for clinical use, which is in line with commonly applied thresholds in the research [22].

To assess interversion agreement, we calculated paired between-version mean differences, confidence intervals (CIs), and the standard error of measurement (SEM) to quantify the level of agreement. We compared SEM values to the minimum important change (MIC) of the PRWE, as defined by Hoogendam et al. [7], to determine whether the SEM exceeded the threshold for clinical relevance (PRWE total score = 20, PRWE pain subscore = 10, PRWE hand function subscore = 11) [7]. Additionally, we created Bland-Altman plots to visualize and further evaluate the level of agreement. The plots show the between-version mean difference, the individual score differences, and the 95% limits of agreement, which indicate the range within which approximately 95% of the individual differences between measurements are expected to fall.

To explore potential gender-related differences, sensitivity analyses were performed stratified by gender and comparing interversion reliability and agreement parameters between men and women using 95% CIs. Independent t-tests were used to compare mean PRWE scores, mean DT-PRWE scores, and between-version mean differences between genders. Finally, we calculated the median (IQR) completion time and compared completion efficiency between versions using the paired Wilcoxon signed-rank test.

Of the 427 patients who completed the PRWE, 45% (192) did not complete the DT-PRWE and therefore were considered missing. To evaluate the nature of the missing data, we conducted a nonresponder analysis using independent t-tests, chi-square tests, and a Mann-Whitney U test to test for differences in demographic variables and PRWE scores. In addition to p values, we calculated standardized mean differences (SMDs) to assess the magnitude of between-group differences. This analysis showed that nonresponders were younger, with a mean ± SD age of 47 ± 18 compared with 53 ± 17 for responders (SMD 0.33; p = 0.006). Nonresponders differed in hand dominance (SMD 0.29; p = 0.04) and received less surgical treatment: 38% (73 of 192) of patients were treated surgically among nonresponders versus 61% (72) among responders (SMD 0.46; p < 0.001). No significant clinically relevant differences were found between responders and nonresponders for the PRWE total score, pain subscore, function subscore, or other patient characteristics. However, SMDs > 0.20 were observed for education level (SMD 0.29) and diagnosis (SMD 0.44). These differences reflected distributional variation across multiple small categories rather than a clinically meaningful imbalance (Supplemental Table 1; http://links.lww.com/CORR/B519). Additionally, we performed a Little test to assess the null hypothesis that the DT-PRWE scores were missing completely at random [15]. The nonsignificant result of the Little test (p = 0.20) indicated that the missing values were not systematically related to the measured patient or clinical characteristics and therefore were unlikely to bias our study results. We performed all analyses using R, version 4.4.1, with the RStudio interface.

## Results

### Interversion Reliability and Interversion Agreement

**Fig. 3:**
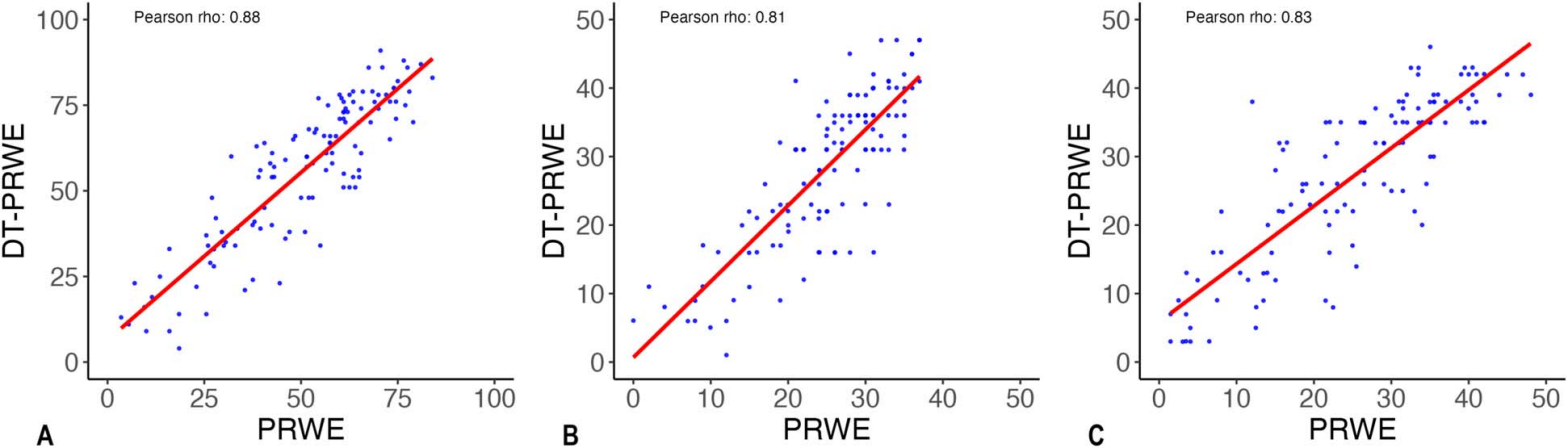
Scatter plots demonstrating the correlation between the (**A**) DT-PRWE and PRWE for the total score, (**B**) pain subscore, and (**C**) hand function subscore. Each dot represents the paired DT-PRWE and PRWE scores for an individual patient.

We observed good interversion reliability between the DT-PRWE and the PRWE across all scores: total score (ICC 0.88 [95% CI 0.83 to 0.91]), pain subscore (ICC 0.78 [95% CI 0.69 to 0.84]), and hand function subscore (ICC 0.83 [95% CI 0.77 to 0.88]) (Table 2). In addition, the Pearson correlation coefficients between the PRWE and the DT-PRWE were also high: r = 0.88 (95% CI 0.83 to 0.92) for the total score, r = 0.81 (95% CI 0.74 to 0.87) for the pain subscore, and r = 0.83 (95% CI 0.77 to 0.88) for the hand function subscore (Fig. 3).

**Table 2.** Levels of reliability and agreement between full-length and decision tree PRWE (n = 119)

Measure	ICC (95% CI)	Pearson (95% CI)	SEM
Total score	0.88 (0.83-0.91)	0.88 (0.83-0.92)	7.1
Pain subscore	0.78 (0.69-0.84)	0.81 (0.74-0.87)	4.6
Hand function subscore	0.83 (0.77-0.88)	0.83 (0.77-0.88)	4.9

Interversion reliability was interpreted using an ICC threshold of ≥ 0.75, indicating acceptable reliability for clinical use. Additionally, Pearson correlation coefficients ≥ 0.70 are considered acceptable for clinical use. Agreement was quantified using the SEM and interpreted relative to the MIC. MIC thresholds for clinical difference are 20 for total, 10 for pain, and 11 for hand function. An SEM below the MIC denotes no measurable clinical difference.

**Fig. 4:**
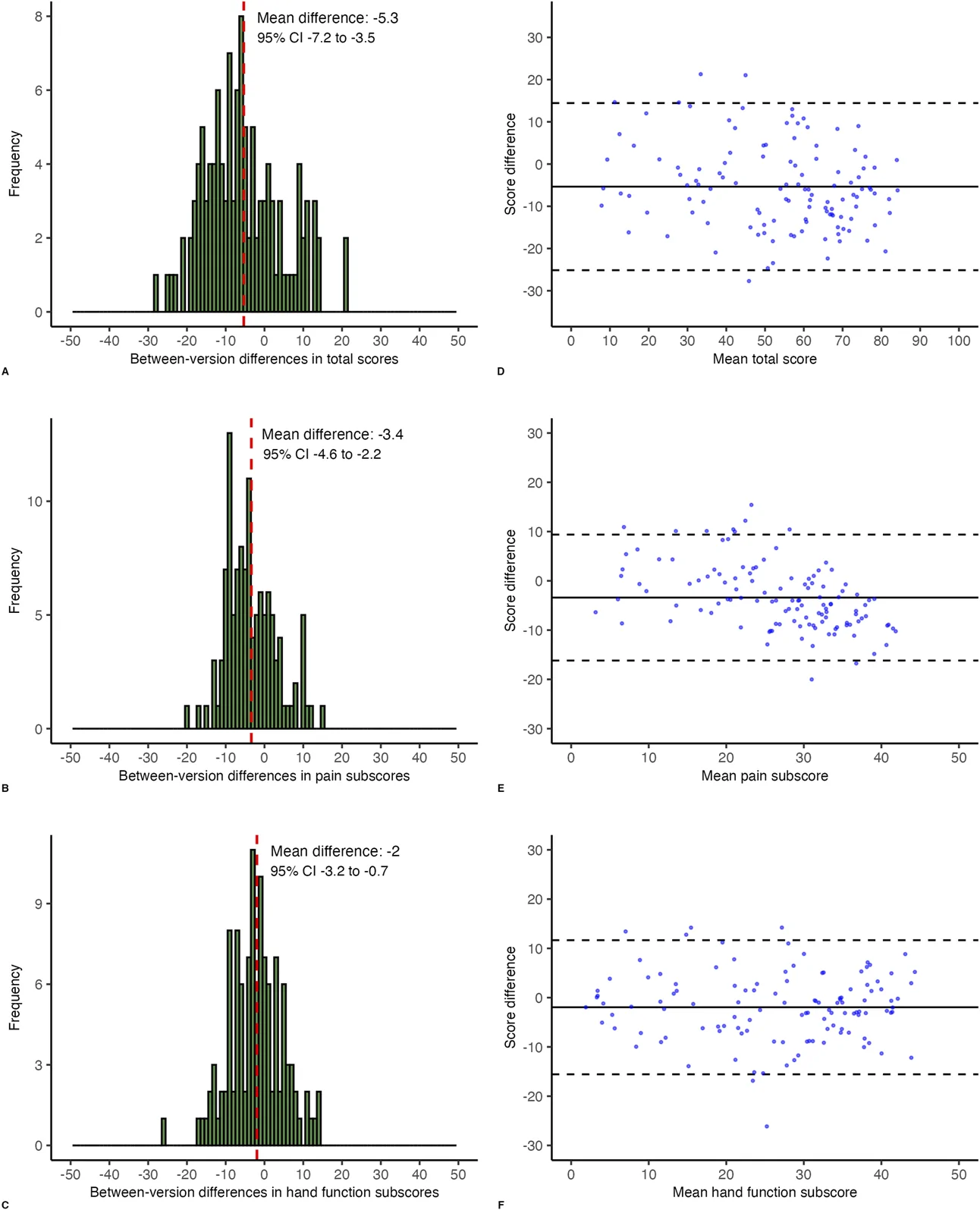
(**A-C**) Distribution of score differences and (**D-F**) Bland-Altman plots between the DT-PRWE and PRWE for (**A, D**) total score, (**B, E**) pain subscore, and (**C, F**) hand function subscore. In the histograms, the dashed red lines represent the between-version mean difference. In the Bland-Altman plots, the solid black lines represent the between-version mean difference, and the dashed black lines represent the lower and upper limits of agreement. Each dot represents the score difference for an individual patient. A color image accompanies the online version of this article.

Similarly, we found high agreement between both versions across all scores, with SEM values of 7.1 (total score), 4.6 (pain subscore), and 4.9 (hand function subscore) (Table 2). All SEM values remained below the MIC of the PRWE. The differences between the outcomes of the PRWE and the DT-PRWE followed a normal distribution (Table 3), with between-version mean differences of -5.3 (95% CI -7.2 to -3.5) on the total score (range 0 to 100) (Fig. 4A), -3.4 (95% CI -4.6 to -2.2) on the pain subscore (range 0 to 50) (Fig. 4B), and -2.0 (95% CI -3.2 to -0.7) on the hand function subscore (range 0 to 50) (Fig. 4C). Additionally, the Bland-Altman plots indicated high agreement, with between-version mean differences close to 0 and differences evenly distributed across the score range for total score (Fig. 4D), pain subscore (Fig. 4E), and hand function subscore (Fig. 4F).

**Table 3.** Comparison of scores and completion time between the PRWE and DT-PRWE

Measure	PRWE	DT-PRWE	Mean difference (95% CI) or median difference (p value)
Total PRWE score (0 to 100)	50.4 ± 19.2	55.7 ± 21.3	-5.3 (-7.2 to -3.5)
Pain subscore (0 to 50)	24.9 ± 8.2	28.3 ± 11.1	-3.4 (-4.6 to -2.2)
Hand function subscore (0 to 50)	25.5 ± 12.0	27. 4 ± 12.1	-2.0 (-3.2 to -0.7)
Completion time in seconds	213 (140 to 438)	65 (50 to 89)	148 (p < 0.001)

PRWE scores are presented as mean ± SD and compared using paired between-version mean differences with 95% CIs. Completion times are presented as median (IQR), with the between-version median difference reported; paired comparisons were performed using the Wilcoxon signed-rank test, and the corresponding p value is shown.

In a gender-based sensitivity analysis, we found no meaningful differences in interversion reliability and agreement between men and women across outcomes. Between-version mean differences were similar across genders, and SEM values in both groups remained below the MIC thresholds, which indicated no clinically relevant measurement differences (Supplemental Table 2; http://links.lww.com/CORR/B519).

### Completion Time

The median (IQR) time to complete the PRWE was 3 minutes 33 seconds (2 minutes 20 seconds to 7 minutes 18 seconds), whereas the median completion time for the DT-PRWE was 1 minute 5 seconds (50 seconds to 1 minute 29 seconds), representing a 70% reduction in time (Table 3). A demonstration version of the DT-PRWE is available online (https://doi.org/10.6084/m9.figshare.31211353) in both English and Dutch. We have also made the conditional questionnaire structure available online (https://doi.org/10.6084/m9.figshare.31211473). The questionnaire structure is provided as both an .xlsx file, which can be used to implement the decision-tree logic in other PROM systems, and an .lss file, which can be directly uploaded to LimeSurvey to deploy the DT-PRWE.

## Discussion

The PRWE is a commonly used PROM in hand and wrist care to assess pain and hand function; however, its length may limit response rates in routine clinical practice. The DT-PRWE was developed to reduce respondent burden by shortening the instrument from 15 to 6 items. Although the DT-PRWE demonstrated good interversion reliability and high agreement with the PRWE in internal validation using simulated data, its clinical usability had not yet been evaluated. External validation in a clinical setting was therefore required to determine whether these measurement properties are maintained in everyday care. We found that the DT-PRWE has comparable interversion reliability and agreement with the PRWE in clinical practice while reducing completion time by 70%. This suggests that the DT-PRWE can be used in routine care to efficiently assess pain and hand function without compromising measurement quality. Clinicians and researchers may consider adopting the DT-PRWE in settings where minimizing patient burden is essential, particularly when multiple PROMs are administered.

### Limitations

An important limitation of our study was the relatively high nonresponse rate to the DT-PRWE. Because it was added to existing digital routine outcome measures and completion of the DT-PRWE was voluntary and not required for clinical care, some patients may have perceived the second questionnaire as optional or redundant, despite its short completion time. This likely contributed to the substantial nonresponse rate and may have introduced selection bias. To address this, we conducted an extensive nonresponder analysis. Although nonresponders were younger, had different hand dominance, and were less likely to have undergone surgical treatment, no significant or clinically relevant differences in PRWE scores were found between responders and nonresponders. In addition, the nonsignificant result of the Little test suggests that the missing DT-PRWE data were missing completely at random and were unlikely to be systematically related to measured patient or clinical characteristics. Taken together, these findings suggest that while nonresponse was substantial, it is unlikely to have meaningfully biased the observed agreement and reliability between the DT-PRWE and the PRWE.

Another limitation of adding the DT-PRWE to the routine outcome measure was that the DT-PRWE was completed after the PRWE. Methodologically, it was preferable to complete the DT-PRWE before the PRWE; this limits the risk of recall bias and learning effects, as not all questions are asked on the DT-PRWE. To reduce these recall and learning effects, we used a 5- to 10-day interval between completing the two versions, a common strategy in test-retest studies to improve measurement independence. Additionally, to minimize changes attributed to clinical interventions, patients who received any treatment or had an additional clinical visit between questionnaire administrations were excluded. Together, these measures were intended to enhance comparability between administrations and support the validity of the observed reliability and agreement between versions.

In addition, within our routine outcome collection, the PRWE was administered only to patients with wrist conditions. Although the PRWE has been validated for other hand-related conditions [30], our findings validate the DT-PRWE specifically for wrist-related diagnoses. Therefore, it remains uncertain whether the DT-PRWE is also valid for broader hand conditions. However, the PRWE is specifically recommended by ICHOM for wrist conditions [29]. In contrast, other PROMs, such as the Michigan Hand Questionnaire [3] and the Boston Carpal Tunnel Questionnaire [14], are recommended for other hand-related conditions [29]. Furthermore, DT-PRWE development was based on the Dutch version of the PRWE [26], which was also used in this study. Cross-cultural validation of the DT-PRWE therefore may be needed to assess its validity in other languages. Finally, our study only investigated interversion reliability and agreement. The test-retest reliability of the DT-PRWE was not evaluated and thus remains unknown.

### Discussion of Key Findings

Our study demonstrated good interversion reliability between the DT-PRWE and PRWE across all scores. Compared with the validation data set used in the development study by Van der Oest et al. [26], this study showed a slight decrease in ICC values. This decline is in line with external validation generally yielding lower correlations than internal validation, in which DT-PRWE scores were simulated from PRWE responses and then compared with the same instrument, reducing interinstrument variability. In contrast, our study used a 5- to 10-day interval between administrations to minimize memory effects from previous responses. Although this approach improves measurement independence, such intervals may also cause natural response variability. Nonetheless, our results were comparable to the test-retest reliability of the PRWE [17, 30], indicating that interversion reliability between the DT-PRWE and the PRWE is comparable to the stability of repeated PRWE measurements over time.

Similarly, we found high agreement between versions across all scores. Although a small directional shift was observed in the between-version mean differences and their CIs, with DT-PRWE scores slightly higher on average, the magnitude of these between-version mean differences and the SEM values did not exceed the MIC thresholds. Moreover, the SEM values we found aligned with those reported in previous test-retest studies on the PRWE [9, 18, 21].

Given these results, our findings highlight the potential of the DT-PRWE in daily clinical care. A key practical advantage of the DT-PRWE is a substantial reduction in patients’ burden by significantly shortening completion time compared with the PRWE [1, 5]. Although the absolute time saved may appear modest, PROMs such as the PRWE are rarely administered in isolation and are commonly combined with multiple outcome measures in routine care [29]. In this context, reducing completion time per PROM may meaningfully decrease overall respondent burden, which is known to negatively affect compliance with PROM completion [1]. Future research may evaluate whether implementation of the DT-PRWE, alone or in combination with other shortened PROMs, improves PROM completion rates in clinical practice.

In this broader context of PROM burden reduction, more sophisticated computer-adaptive tests (CATs) based on item response theory (IRT), such as those in the Patient-Reported Outcomes Measurement Information System (PROMIS) framework, also aim to reduce burden [2, 4], with completion times per subdomain comparable to those of the DT-PRWE [10].

IRT-based CATs are theoretically advantageous because they may further optimize precision for some patients by tailoring the number of items to individual response patterns [2, 4]. However, their implementation requires calibrated item banks and dedicated software infrastructure, which can limit feasibility in routine clinical practice [2, 4]. In contrast, the DT-PRWE’s fixed structure enables relatively simple implementation in standard digital PROM collection systems. It supports conditional item routing without the need for an item bank, extensive calibration, or costly software, making it a more practical alternative to the more sophisticated IRT-based CATs in many clinical settings [2, 4].

An additional advantage is that the DT-PRWE maintains direct comparability with existing PRWE data, avoiding a break in ongoing clinical and research data sets. Moreover, as demonstrated in internal validation, the DT-PRWE total score [26] showed comparable outcomes on a similar scale (score range 0 to 100) with IRT-based versions of the 11-item Patient Evaluation Measure for thumb base osteoarthritis [11] and cubital tunnel syndrome [25], which supports its use as a suitable and easy-to-implement alternative to IRT-based CATs. The demonstration version and questionnaire structure we provided (https://doi.org/10.6084/m9.figshare.31211473) may support this implementation.

### Conclusion

In this study, we found that the DT-PRWE is a reliable alternative to the PRWE, requiring substantially less time for patients to complete. By preserving both pain and function subscores and reducing the number of questions, the DT-PRWE offers a practical way to assess wrist-related outcomes with less burden for patients. Clinicians and researchers can use the DT-PRWE as a direct substitute for the PRWE in routine care and research settings. Its simple digital implementation and comparability with existing PRWE data make it well suited to support more efficient, patient-centered, value-based care without disrupting ongoing outcome monitoring. Future studies should focus on cross-cultural validation of the DT-PRWE and on evaluating test-retest reliability to further establish its measurement properties. In addition, prospective implementation studies are needed to determine whether the use of shortened PROMs such as the DT-PRWE leads to higher completion rates and improved compliance in clinical practice.

## Group Authors

Members of the Hand-Wrist Study Group include: Richard Arjen Michiel Blomme, Jeronimus (Jeroen) Maria Smit, Kennard Harmsen, Gertjan Halbesma, Guus Maarten Vermeulen, Johannes (Hans) Pieter de Schipper, Jeroen Hein van Uchelen, Oliver Theodor Zöphel, John Sebastiaan Souer, Lisa Esteban Lopez, Alexandra Fink, Rob van Huis, Pierre-Yves Alain Adriaan Pennehouat, Karin Schoneveld, Grada Renée Arends, Reinier Feitz, Lisa Hoogendam, Steven Eric Ruden Hovius, Yara Eline van Kooij, Jaimy Emerentiana Koopman, Mark Johannes Willem van der Oest, Willemijn Anna de Ridder, Ruud Willem Selles, Liz-Tipper Sikking, Harm Pieter Slijper, Marloes Hendrina Paulina ter Stege, Joris Sebastiaan Teunissen, Robbert Maarten Wouters, Nina Louisa Loos, Nienke Helena Adriana Mendelaar, Lyse van Wijk, Ward Rogier Bijlsma, Joost W. Colaris, Liron S. Duraku, Egberta Petronella, Adriana (Brigitte) van der Heijden, Caroline Anna Hundepool, Jelle Michiel Zuidam.
